# Walking a tightrope, teetering on the edge, fighting demons: women’s experiences of having an eating disorder while pregnant

**DOI:** 10.1186/2050-2974-2-S1-O51

**Published:** 2014-11-24

**Authors:** Terri Burton

**Affiliations:** 1 University of Notre Dame Australia, Fremantle, Australia

## 

Whilst extant literature demonstrates the aetiology, incidence and outcomes of eating disorders in women, with evidence showing the impacts on pregnancy, the fetus and parenthood adjustment, there is a paucity of research regarding how women make meaning of the experience. A phenomenological study provided a description of the pregnancy experience as perceived by a cohort of women with diagnosed eating disorders. Data were collected via semi structured interviews with women with a known diagnosis of an eating disorder either in the past or present and who had been pregnant within the previous twelve months. Data were analysed using constant comparative approach seeking themes and patterns.

The study identified fourteen key themes that describe the women's experience of living with an eating disorder while pregnant. These themes included a basic description of the pregnancy journey itself; the use of metaphor; perceptions of motherhood; concerns about the baby; and body image. In addition, eating disorder behaviour; eating disorder status; secrets; wishes; misconceptions; support; emotions; health professionals' interactions and healthcare improvements were also identified. It is important that healthcare providers have an awareness that pregnant women may be undergoing personal distress and to be sensitive to the specific needs of women with eating disorders. This study has raised awareness of this issue and provided a baseline for further research in the area.

This abstract was presented in the **Learning from Consumers** stream of the 2014 ANZAED Conference.

